# Iron-Hepcidin Dysmetabolism, Anemia and Renal Hypoxia, Inflammation and Fibrosis in the Remnant Kidney Rat Model

**DOI:** 10.1371/journal.pone.0124048

**Published:** 2015-04-13

**Authors:** Patrícia Garrido, Sandra Ribeiro, João Fernandes, Helena Vala, Elsa Bronze-da-Rocha, Petronila Rocha-Pereira, Luís Belo, Elísio Costa, Alice Santos-Silva, Flávio Reis

**Affiliations:** 1 Laboratory of Pharmacology & Experimental Therapeutics, Institute for Biomedical Imaging and Life Sciences (IBILI), Faculty of Medicine, University of Coimbra, Coimbra, Portugal; 2 Center for Neuroscience and Cell Biology—Institute for Biomedical Imaging and Life Sciences (CNC.IBILI) Research Unit, University of Coimbra, Coimbra, Portugal; 3 Research Unit on Applied Molecular Biosciences (UCIBIO), REQUIMTE, Department of Biological Sciences, Laboratory of Biochemistry, Faculty of Pharmacy, University of Porto, Porto, Portugal; 4 Center for Studies in Education, Technologies and Health (CI&DETS), Agrarian School of Viseu, Polytechnic Institute of Viseu, Viseu, Portugal; 5 Research Centre in Health Sciences, University of Beira Interior, Covilhã, Portugal; Lady Davis Institute for Medical Research/McGill University, CANADA

## Abstract

Anemia is a common complication of chronic kidney disease (CKD) that develops early and its severity increases as renal function declines. It is mainly due to a reduced production of erythropoietin (EPO) by the kidneys; however, there are evidences that iron metabolism disturbances increase as CKD progresses. Our aim was to study the mechanisms underlying the development of anemia of CKD, as well as renal damage, in the remnant kidney rat model of CKD induced by 5/6 nephrectomy. This model of CKD presented a sustained degree of renal dysfunction, with mild and advanced glomerular and tubulointerstitial lesions. Anemia developed 3 weeks after nephrectomy and persisted throughout the protocol. The remnant kidney was still able to produce EPO and the liver showed an increased *EPO* gene expression. In spite of the increased EPO blood levels, anemia persisted and was linked to low serum iron and transferrin levels, while serum interleukin (IL)-6 and high sensitivity C-reactive protein (hs-CRP) levels showed the absence of systemic inflammation. The increased expression of duodenal ferroportin favours iron absorption; however, serum iron is reduced which might be due to iron leakage through advanced kidney lesions, as showed by tubular iron accumulation. Our data suggest that the persistence of anemia may result from disturbances in iron metabolism and by an altered activity/function of EPO as a result of kidney cell damage and a local inflammatory milieu, as showed by the increased gene expression of different inflammatory proteins in the remnant kidney. In addition, this anemia and the associated kidney hypoxia favour the development of fibrosis, angiogenesis and inflammation that may underlie a resistance to EPO stimuli and reduced iron availability. These findings might contribute to open new windows to identify putative therapeutic targets for this condition, as well as for recombinant human EPO (rHuEPO) resistance, which occurs in a considerable percentage of CKD patients.

## Introduction

Chronic kidney disease (CKD) is a pathological condition that results from a gradual and permanent loss of kidney function over time, usually, months to years. CKD can result from primary diseases of the kidneys, however, diabetic nephropathy and hypertension are the main causes of CKD [1]. Anemia is a common complication of CKD that develops early in the course of the disease, increasing its frequency and severity with the decline of renal function. The incidence of anemia is less than 2% in CKD stages 1 and 2, about 5% in CKD stage 3, 44% in CKD stage 4 and more than 70% in end-stage renal disease (ESRD) [2]. This condition is associated with a decreased quality of life [3], increased hospitalizations [4,5], cardiovascular complications—angina, left ventricular hypertrophy (LVH) and chronic heart failure—and mortality [6–9].

Anemia is mainly associated with a reduced production of erythropoietin (EPO) by the kidneys. However, there are also evidences that iron metabolism disturbances increase as the CKD progresses. The reasons for this high proportion of CKD patients with iron disturbances are not well clarified; however, inflammation has been proposed to play an important role. In fact, previous works reported that ESRD patients under hemodialysis present higher hepcidin serum levels, increased markers of inflammation [such as C-reactive protein (CRP) and interleukin (IL)-6] and reduced iron absorption and mobilization, thus presenting lower levels of iron and transferrin [10–12].

Hepcidin plays pivotal role in the development of the anemia associated with CKD [10]. Hepatocytes play a dual role in iron metabolism, acting as the major site of iron storage and of secretion of the iron regulatory hormone hepcidin (codified by the gene *Hamp*) [13]. Hepcidin orchestrates systemic iron fluxes by controlling iron absorption through enterocytes and iron mobilization from macrophages. Hepcidin binds to the iron exporter ferroportin (SLC40A1, solute carrier family 40, member 1) on the surface of iron-releasing cells, triggering its degradation and, therefore, reducing the iron absorption and mobilization through the linkage of iron to transferrin [13]. The expression of *Hamp* is regulated by different hepatocyte cell-surface proteins, namely hemochromatosis (Hfe), transferrin receptor protein 2 (TfR2), hemojuvelin (HJV), serine protease matriptase-2 (TMPRSS6) and IL-6, and increases in inflammatory conditions (through IL-6 dependent pathway), in increased erythropoiesis and iron overload, and is down-regulated during hypoxia or iron deprivation [14].

During the last few years the mechanisms underlying hepcidin and iron regulation have been largely studied. In addition, the impact of renal hypoxia, through hypoxia-inducible factors (HIFs), on iron metabolism, on kidney lesion or regeneration, as well as on hepcidin expression, have been extensively debated [15–17]. In response to low oxygen supply, HIFs are produced, triggering the expression of the hypoxia response genes, leading to an increased production of EPO, vascular endothelial growth factor (VEGF) and glycolytic enzymes [18].

Experimental models using transgenic mice, knockout for some of the key mediators, have been crucial to reveal some of these new findings [19,20]. Uremic rat models have been characterized and used for long time by our group, as well as by other authors, as tools to study the pathophysiological events underlying kidney disease development; renal failure was induced in these uremic rat models by nephrectomy and infarction [21–23]. However, the information is still scarce concerning the characterization of iron dysfunction associated with hypoxic anemia of chronic kidney disease, namely in the 5/6 nephrectomized rat, which is one of the most used rat model of CKD. In this sense, we intended to elucidate the mechanisms underlying the development of anemia and evolution of renal damage in the remnant kidney rat model of CKD induced by 5/6 nephrectomy, focusing on iron impairment and kidney hypoxia, inflammation and fibrosis.

## Material and Methods

### Animals and experimental protocol

Male Wistar rats (Charles River Lab., Inc., Barcelona, Spain) weighing 300g were maintained in an air conditioned room, subjected to 12 h dark/light cycles and given standard rat diet (IPM-R20, Letica, Barcelona, Spain) *ad libitum* and free access to tap water. Animal experiments were conducted according to the European Communities Council Directives on Animal Care. The experiments received the approval by the Portuguese Foundation for Science and Technology and the Local Ethics Committee of the Faculty of Medicine from the University of Coimbra.

The rats were divided into two groups (7 rat each): Sham group—subjected to chirurgical process but without kidney mass reduction and chronic renal failure (CRF) group—induced by a two-stage (5/6) nephrectomy, with subtraction firstly of about 2/3 of the left kidney by left flank incision and, one week later, complete removal of the right kidney through identical incision/procedure. All the animals have completed 12 weeks of protocol. Body weight (BW) was monitored throughout the study and blood pressure (BP) and heart rate (HR) measures were obtained using a tail-cuff sphygmomanometer LE 5001 (Letica, Barcelona, Spain).

### Sample collection and preparation

At the beginning of the experiments (T0) and at 3 (T1), 6 (T2) 9 (T3) and 12 (T4) weeks after the surgical 5/6 nephrectomy, the rats were subjected to intraperitoneal anesthesia with a 2 mg/kg BW of a 2:1 (v:v) 50 mg/mL ketamine (Ketalar, Parke-Davis, Lab. Pfeizer Lda, Seixal, Portugal) solution in 2.5% chlorpromazine (Largactil, Rhône-Poulenc Rorer, Lab. Vitória, Amadora, Portugal), to collect blood by venipuncuture, from the jugular vein, into vacutainer tubes without anticoagulant (to obtain serum) or with K_3_EDTA for hematological and biochemical studies; at T0, T1, T2 and T3 a small blood was collected to monitor anemia and renal function; at the end of protocol (T4) 10 mL of blood were collected, to perform all the biochemical and hematological assays.

At the end of the protocol, after collection of blood, the rats were sacrificed by cervical dislocation; kidneys, duodenum, liver and heart were immediately removed, placed in ice-cold Krebs-Henseleit buffer and carefully cleaned. A bone marrow aspirate from the femur was also performed.

### Biochemical and hematological assays

Serum creatinine and blood urea nitrogen (BUN) were used as renal function markers; glicose, total cholesterol (Total-c), triglycerides (TGs), creatine kinase (CK), aspartate transaminase (AST) and alanine transaminase (ALT) were analysed through automatic validated methods and equipments (Hitachi 717 analyser, Roche Diagnostics Inc., Massachuasetts, USA).

Red blood cells (RBC) count, hematocrit (Hct), hemoglobin (Hb), reticulocyte count (Ret) mean cell hemoglobin (MCH), mean cell hemoglobin concentration (MCHC), mean cell volume (MCV), platelet count (PLT), platelet distribution width (PDW), RBC distribution width (RDW) and white blood cells (WBC) were assessed in whole blood K_3_EDTA (Coulter Counter, Beckman Coulter, Inc., Fullerton, California, USA).

Serum iron concentration was determined using a colorimetric method (Iron, Randox Laboratories Ltd., North Ireland, UK), whereas serum ferritin and transferrin were measured by immunoturbidimetry (Laboratories Ltd., North Ireland, UK).

Serum levels of interleukin-6 (IL-6), interferon *γ* (IFN-*γ*), transforming growth factor (TGF-β1) and vascular endothelial growth factor (VEGF) were all measured by rat-specific Quantikine ELISA kits from R&D Systems (Minneapolis, USA). High-sensitive C-reactive protein (hs-CRP) was determined by using a rat-specific Elisa kit from Alpha Diagonostic International (San Antonio, USA). Serum levels of erythropoietin (EPO) were evaluated by rat specific ELISA kit (MyBioSource, USA).

Quantification of total bilirubin was performed by a colorimetric test of diazotized sulfanilic acid reaction (Roche Diagnostics Inc., Massachusetts, USA) circulating levels of glucose and uric acid were determined by routine automated technology (ABX Diagnostics, CA, USA).

### Flow cytometry

To study leukocyte activation, the mononuclear cells were then isolated from the other blood cells by density gradient centrifugation (700g, 20 min. at room temperature) (Histopaque-1077 and -1119, from Sigma-Aldrich, Sintra, Portugal) followed by 3 washing steps with phosphate buffered saline solution (PBS, pH = 7.4), supplemented with 3% (v/v) fetal bovine serum (FBS). We used antibodies against CD3, CD4, CD8 and CD25 (all from BD Biosciences, San Diego, CA, USA), conjugated either to FITC, PE or PerCP. Fluorochrome-conjugated isotype-matched antibodies were used as negative controls. For surface staining, mononuclear cells (≈1 × 10^6^ cells in 100 μl PBS containing 3% (v/v) fetal bovine serum and 0.1% NaN_3_) were incubated with 1 μg antibody in the dark, at room temperature, for 30 min and then washed three times with PBS supplemented with FBS 3% (v/v); 400 μl of PBS supplemented with 3% (v/v) FBS was finally added to each tube. The treated samples and controls were analyzed by flow cytometry within a 1 h period. Flow cytometric analysis was carried out in a FACS Calibur (San Jose, CA, USA) based on the acquisition of 20000 events. Detectors for forward (FSC) and side (SSC) light scatter were set on a linear scale, whereas logarithmic detectors were used for all three fluorescence channels (FL-1, FL-2 and FL-3). Compensation for spectral overlap between FL channels was performed for each experiment using single-color-stained cell populations. For the experimental samples, a corresponding isotype control was used to set gates, or positive/negative cell populations. All data were analyzed using FlowJo software (TreeStar Inc, Ashland, OR, USA).

### Gene expression analysis

In order to isolate total RNA, 0.2 g samples of liver, duodenum and kidney, from each rat, were immersed in RNA laterTM (Ambion, Austin, USA) upon collection and stored at 4°C for 24h; afterwards, samples were frozen at -80°C. Subsequently, tissue samples weighing 50±10 mg were homogenized in a total volume of 1 ml TRI Reagent (Sigma, Sintra, Portugal) using a homogenizer, and total RNA was isolated as described in the TRI Reagent Kit. To ensure inactivation of contaminating RNAses, metal objects and glassware were cleaned with detergent, immersed in RNAse-free water (0.2% diethyl pyrocarbonate) for 2 h and finally heated at 120°C for 1 h. RNA integrity (RIN, RNA Integrity Number) was analyzed using 6000 Nano ChipW kit, in Agilent 2100 bioanalyzer (Agilent Technologies, Walbronn, Germany) and 2100 expert software, following manufacturer instructions. The yield from isolation was from 0.5 to 1.5 μg; RIN values were 7.0–9.0 and purity (A260/A280) was 1.8–2.0. The concentration of the RNA preparations were confirmed with NanoDrop1000 (ThermoScientific,Wilmington, DE, USA). Possible contaminating remnants of genomic DNA were eliminated by treating these preparations with deoxyribonuclease I (amplification grade) prior to RT-qPCR amplification. Reverse transcription and relative quantification of gene expression were performed as previously described [24]. Real-time qPCR reactions were performed for the following genes: *EPO*, *EPOR*, Transferrin receptor 2 (*TfR2*), Hepcidin (*Hamp*), Ferroportin (*SLC40A1*), Hemojuvelin (*HJV*), Transferrin (*TF*), Hemochromatosis (*Hfe*), +IRE-Divalent Metal Transporter 1 (*DMT1*), Transferrin recptor 1 (*TfR1*), Matriptase-2 (*TMPRSS6*), Interleukin-6 (*IL-6*) and Bone Morphogenic Protein 6 (*BMP6*); vascular endothelial growth factor *(VEGF)*, interleukin-1 beta *(IL-1β)*, nuclear transcription factor kappa B (*NF-kB)*, connective tissue growth factor (*CTGF)* and tumor necrosis factor alpha *(TNF-α)* which were normalized in relation to the expression of beta-actin (*Actb*), and 18S ribosomal subunit (*18S*). Primer sequences are listed on Table 1. Results were analyzed with SDS 2.1 software (Applied Biosystems, Foster City, CA, USA) and relative quantification calculated using the 2^-ΔΔCt^ method [25]. In liver tissue we studied the *EPO*, *EPOR*, *TfR1*, *TfR2*, *Hamp*, *Il-6*, *SLC40A1*, *HJV*, *TF*, *Hfe*, *BMP6* and *TMPRSS6* gene expression; in duodenum tissue the gene expression of *DMT1* and *SLC40A1* were studied, and in the kidney we evaluated the expression of *EPO*, *EPOR*, *Il-6*, *IL-1β*, *TNF-α*, *NF-kB*, *CTGF and VEGF* genes.

**Table 1:** List of primer sequences (F: forward; R: reverse).

Gene	Primer sequences
**EPO**	F: 5’-AGGGTCACGAAGCCATGAAG-3’
	R: 5’-GAT TTC GGC TGT TGC CAG TG-3’
**EPOR**	F: 5’-GCG ACT TGG ACC CTC TCA TC-3’
	R: 5’-AGT TAC CCT TGT GGG TGG TG-3’
**Hamp**	F: 5’-GAA GGC AAG ATG GCA CTA AGC-3’
	R: 5’-CAG AGC CGT AGT CTG TCT CG-3’
**TfR2**	F: 5’-CAA GCT TCG CCC AGA AGG TA-3’
	R: 5’-CGT GTA AGG GTC CCC AGT TC-3’
**SLC40A1**	F: 5’-CAG GCT TAG GGT CTA CTG CG-3’
	R: 5’-CCG AAA GAC CCC AAA GGA CA-3’
**HJV**	F: 5’-GCC TAC TTC CAA TCC TGC GT-3’
	R: 5’-GGT CAA GAA GAC TCG GGC AT-3’
**TF**	F: 5’-GGC ATC AGA CTC CAG CAT CA-3’
	R: 5’-GCA GGC CCA TAG GGA TGT T-3’
**Hfe**	F: 5’-CTG GAT CAG CCT CTC ACT GC-3’
	R: 5’-GTC ACC CAT GGT TCC TCC TG-3’
**DMT1**	F: 5’-CAA CTC TAC CCT GGC TGT GG-3’
	R: 5’-GTC ATG GTG GAG CTC TGT CC-3’
**TfR1**	F: 5’-GCT CGT GGA GAC TAC TTC CG-3’
	R: 5’-GCC CCA GAA GAT GTG TCG G-3’
**TMPRSS6**	F: 5’-CCG AAT ATG AGG TGG ACC CG-3’
	R: 5’-GGT TCA CGT AGC TGT AGC GG-3’
**BMP6**	F: 5’-GCT GCC AAC TAT TGT GAC GG-3’
	R: 5’-GGT TTG GGG ACG TAC TCG G-3’
**Il-6**	F: 5’-ATG TTG TTG ACA GCC ACT GC-3’
	R: 5’- TTT TCT GAC AGT GCA TCA TCG-3’
**Il-1β**	F: 5’-CTC TGT GAC TCG TGG GAT GAT G-3’
	R: 5’-CAC TTG TTG GCT TAT GTT CTG TCC-3’
**CTGF**	F: 5’-CGT AGA CGG TAA AGC AAT GG-3’
	R: 5’-AGT CAA AGA AGC AGC AAA CAC-3’
**NF-kβ**	F: 5’-ACC TGA GTC TTC TGG ACC GCT G-3’
	R: 5’-CCA GCC TTC TCC CAA GAG TCG T-3’
**VEGF-α**	F: 5’- GAA GTT CAT GGA CGT CTA CCA G -3’
	R: 5’- CAT CTG CTA TGC TGC AGG AAG CT -3’
**TNF-α**	F: 5’- CCC AGA CCC TCA CAC TCA GAT CAT -3’
	R: 5’—GCA GCC TTG TCC CTT GAA GAG AA-3’
**18S**	F: 5’-CCA CTA AAG GGC ATC CTG GG-3’
	R: 5’-CAT TGA GAG CAA TGC CAG CC-3’
**Actb**	F: 5’-GAG ATT ACT GCC CTG GCT CC-3’
	R: 5’-CGG ACT CAT CGT ACT CCT GC-3’

### Western blot assay

The duodenum proteins were extracted using RIPA buffer. After centrifugation, protein concentration in supernatant was assayed using the bicinchoninic acid (BCA) method (Thermo Scientific Pierce, IL, USA). Aliquots of the extract containing 200 μg of protein were separated by reducing SDS-PAGE (10%) and electroblotted onto nitrocellulose membranes. The blots were blocked by using 7% non-fat milk in a solution of Tris-buffered salt with Tween-20. The blots were incubated with rabbit anti-SLC40A1 antibody (1:100, abcam, Cambridge, UK) overnight at 4°C, then they were incubated in goat-anti-rabbit secondary antibody-conjugated horseradish peroxide (1:1000, SantaCruz Biotechnoloy, TX, USA). Immunoreactive proteins were detected by using the enhanced chemiluminescence method (ECL; WesternBright, Advansta, CA, USA). The blots analysis was performed by densotometry (Bio1D++ version99, Vilber Lourmat). To ensure even loading of the samples, the same membrane was probed with rabbit anti-β-tubulin antibody (SantaCruz Biotechnology, TX, USA) at 1:200 dilution. The protein concentration in each sample was normalized for Sham group.

### Histopathological analysis

Tissue samples were fixed in neutral formalin 10% and embedded in paraffin wax; afterwards, 4*μ*m thick sections for routine histopathological studies were stained with hematoxylin and eosin (H&E). Periodic acid of Shiff (PAS) was used to evaluate and confirm the levels of mesangial expansion, thickening of basement membranes and sclerotic parameters. For PAS staining, the tissue samples were fixed in neutral formalin 10%, embedded in paraffin wax, and 4*μ*m thick sections were immersed in water and subsequently treated with 1% aqueous solution of periodic acid, then washed to remove any traces of the periodic acid, and finally treated with Schiff’s reagent. All samples were examined by light microscopy using a Microscope Zeiss Mod. Axioplan 2. The degree of injury visible by light microscopy was scored in a double-blinded fashion by two pathologists. Lesions were evaluated on the total tissue on the slide. Glomerular and tubulointerstitial lesions were divided in mild and advanced. Mild glomerular damage was assessed by evaluating thickening of Bowman’s capsule, hyalinosis of the vascular pole, glomerular atrophy, hypercellularity and dilatation of Bowman´s space. Advanced glomerular damage was assessed by grading sequentially four main lesions, from the less to the worse one: 1—thickening of glomerular basement membrane (GBM), 2—mesangial expansion, 3—nodular sclerosis and 4—global glomerulosclerosis. When advanced lesions were presented at a given glomeruli, the analysis of mild lesions become unavailable. Mild tubulointerstitial lesions included tubular hyaline droplets, tubular basement membrane (TBM) irregularity, tubular dilatation, interstitial inflammatory infiltration and vacuolar tubular degeneration. Advanced tubulointerstitial lesions were assessed evaluating the presence of hyaline cylinders, tubular calcification, necrosis and the association of interstitial fibrosis and tubular atrophy (IFTA). The evaluation of vascular lesions was focused on arteriolar hyalinosis, arteriolosclerosis and arteriosclerosis.

A semiquantitative rating for each slide ranging from normal (or minimal) to severe (extensive damage) was assigned to each component. Severity of lesions was graded according to the extension occupied by the lesion (% area): 0—absent/normal: 0%; 1 –*<*25%; 2–25–50%; 3 –*>*50%. The final score for each sample was obtained by the average of scores observed in individual glomeruli in the analysed microscopic fields. Tubulointerstitial damage was evaluated and graded by the same semiquantitative method. When using PAS, the rating was set for intensity and extension of staining, ranging from 0 (no staining) to 3 (intense and extensive staining), referring tissue specificity scoring when adequate.

Perl’s Prussian blue staining assay was performed on kidney slides to search for iron accumulation within rat renal tubules.

### Immunohistochemistry analysis

The liver and renal cortex/medulla paraffin sections (4 μm) from each sample were dewaxed in xylene, rehydrated in a series of ethanol washes, and placed in distilled water before staining procedures. The samples were processed for indirect immune detection using a mousse and rabbit specific horseradish peroxidase (HRP)/ diaminobenzidine (DAB) detection IHC kit (ab80436, Abcam Inc, Cambridge, UK), according to the manufactor’s protocol. Negative controls were included in each staining series, by omission of the primary antibodies. Antigen retrieval was performed for 20 min for paraffin-embedded tissue in the preheated Citrate Buffer (10mM Citric Acid, pH 6.0) using a pressure cooker. Between incubations with the antibodies, the specimens were washed two to four times in buffer PBS (pH 7.4). All incubations were performed overnight at 4°C in a humidified chamber. In this study, we employed primary antibodies for detection of hepcidin (dilution; 1:150; ab81010, Abcam Inc., Cambridge, UK), CTGF (dilution 1:250, ab6992; Abcam Inc, Cambridge, UK), NF-κB p50 (dilution 1:500, sc-114; Santa Cruz Biotechnology, Inc.), HIF-1α, (dilution:1:200, sc-53546, Santa Cruz Biotechnology, Inc.), EPAS-1 (H-310) (dilution:1:250, sc-28706, Santa Cruz Biotechnology, Inc.), ARNT1 (dilution:1:100, sc-5580, Santa Cruz Biotechnology, Inc.), ARNT2 (dilution:1:100, sc-5581, Santa Cruz Biotechnology, Inc.). For immunohistochemical quantification, ten 400x microscopic views of liver and renal cortex and medulla per slide were selected randomly and photographed using a Leica DFC480 microscope (Leica Microsystems). Intensity and area of positive staining, detected by brown staining, were used as criteria: intensity was evaluated as weak (1), moderate (2) or strong (3); the percentage of area was quantified. A staining score (Quick Score) was then calculated according to previously described [26], using the formula: Quick Score = intensity (1, 2 or 3) multiplied by area (percentage). The final score (out of maximum of 300) for each group was obtained averaging the individual scores of each animal.

Immunohistochemical studies were evaluated independently by two pathologists blinded to the data. Slight differences in interpretation were resolved by simultaneous viewing.

### Statistical analysis

For statistical analysis, we used the IBM Statistical Package for Social Sciences (SPSS), version 20 (2011). Significance level was accepted at *p* less than 0.05. Results are presented as means ± standard error of means (SEM). Comparisons between groups were performed using non-parametric tests (Mann-Whitney test).

## Results

### Body and tissue weights and blood pressure

At the end of experimental protocol (12 weeks), a significant decrease (p<0.001) of BW was observed in CRF rats, when compared to Sham. In addition, KW and KW/BW (p<0.001 for both) presented higher values in the CRF animals. While HW was unchanged, HW/BW ratio was higher (p<0.01) in CRF rats. Despite the lower value (p<0.01) of LW in the CRF group, LW/BW was higher (p<0.05) when compared with the Sham group (Table 2). CRF rats presented a significantly higher (p<0.01) systolic blood pressure at the final time, when compared with the Sham animals, but similar values were found for DBP, MBP and HR (Table 2).

**Table 2:** Body and tissue weights, blood pressure, biochemical and hematological data at the final time (12 weeks).

Parameters	Sham group	CRF group
**BW (Kg)**	0.45±0.02	0.36±0.01 ***
**KW (g)**	1.22±0.03	1.65±0.04 ***
**KW/BW (g/Kg)**	2.72±0.05	4.61±0.22 ***
**HW (g)**	1.16±0.03	1.24 ± 0.07
**HW/BW (g/Kg)**	2.58±0.08	3.48±0.25 **
**LW (g/Kg)**	13.33±0.48	11.32±0.34 **
**LW/BW (g/Kg)**	29.61±0.65	31.43±0.71 *
**SBP (mmHg)**	117.7 ± 1.15	134.1 ± 4.6 **
**DBP (mmHg)**	112.0 ± 0.58	111.5 ± 4.5
**MBP (mmHg)**	114.3 ± 1.45	121.3 ± 4.5
**HR (beats/min)**	360.7± 1.20	373.0 ± 9.2
**Glicose (mmol/L)**	9.46±0.31	8.66±0.60
**TGs (mmol/L)**	1.05±0.14	1.58±0.32
**Total-c (mmol/L))**	1.25±0.06	2.44±0.54 *
**CK (U/L)**	540.57±58.94	473.00±85.57
**ALT (U/L)**	35.17±2.21	42.00±18.53*
**AST (U/L)**	80.57±7.84	139.43±70.70
**Bilirubin (μmol/L)**	8.04e-5±1.03e-5	1.03e-4±1.71e-5
**hsCRP (μg/mL)**	262.25±12.43	225.31±7.95 *
**INF-γ (pg/mL)**	23.30±3.10	25.51±2.26
**TGF-β1 (ng/mL)**	75.74±5.62	84.13±3.85
**RBC (x 10** ^**12**^ **/L)**	7.94±0.08	6.53±0.43 **
**Ret (x10** ^**9**^ **/L)**	181.22±6.82	168.14±17.32
**MCV (fL)**	52.52±0.53	51.93±0.69
**MCH (pg)**	18.08±0.18	18.36±0.24
**MCHC (g/dL)**	34.60±0.08	35.37±0.19 **
**RDW (%)**	11.48±2.53	18.34±3.23
**PLT (x 10** ^**9**^ **/L)**	713.75±15.19	769.00±73.17
**PDW (%)**	16.34±0.18	16.44±0.20
**WBC (x 10** ^**9**^ **/L)**	1.78±0.30	5.01±1.76
**T Lymphocytes (%)**	57.20±1.36	54.67±2.91
**CD3** ^**+**^ **CD4** ^**+**^ **T cells (%)**	72.18±0.60	72.48±1.16
**CD3** ^**+**^ **CD4** ^**+**^ **CD25** ^**+**^ **T cells (%)**	5.97±0.62	5.85±0.70
**CD3** ^**+**^ **CD8** ^**+**^ **T cells (%)**	24.16±1.34	28.17±2.17
**CD3** ^**+**^ **CD8** ^**+**^ **CD25** ^**+**^ **T cells (%)**	0.40±0.04	0.63±0.01**

Results are presented as mean ± SEM

*- *p* < 0.05

**- *p* < 0.01, and

***- *p* < 0.001 *versus* Sham group.

ALT—alanine transaminase, AST—aspartate transaminase; BW—body weight; CK—creatine kinase; DBP—diastolic blood pressure; Hb—hemoglobin; Hct—hematocrit; HR—heart rate; hsCRP—high-sensitive C reactive protein; HW—heart weight; IFN-*γ* - interferon *γ;* KW, kidney weight; LW, liver weight; MBP, mean blood pressure; MCH—mean cell hemoglobin; MCHC—mean cell hemoglobin concentration; MCV—mean cell volume; PDW—platelet distribution width; PLT—platelets; RBC—red blood cells; RDW—RBC distribution width; Ret—reticulocytes; SBP, systolic blood pressure; TGs—triglycerides; TGF-β1—transforming growth factor beta1; Total-c—total cholesterol; VEGF—vascular endothelial growth factor; WBC—white blood cells.

### Biochemical and hematological data

**Fig 1:**
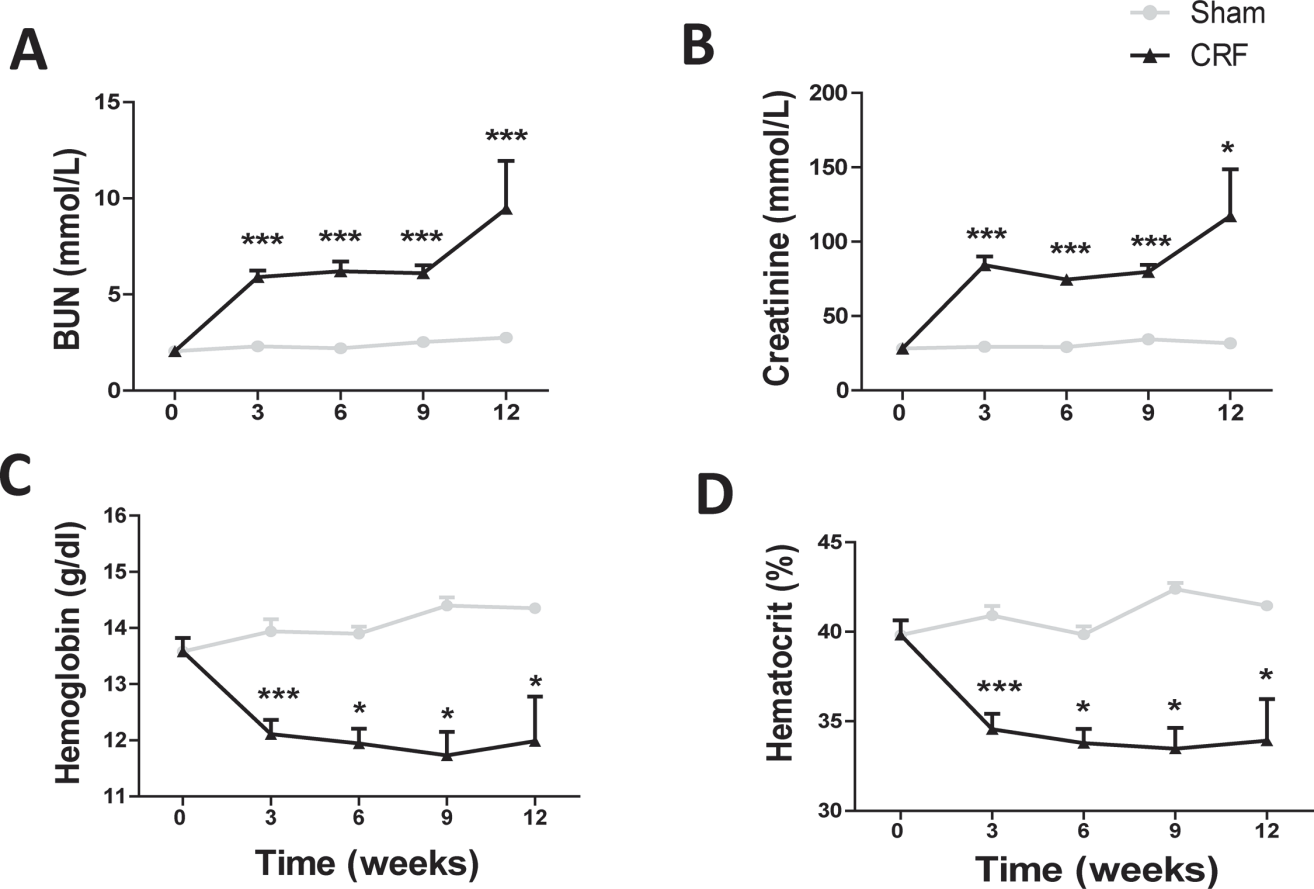
Renal and hematological data throughout the follow-up period of 12 weeks. Evolution of BUN (A), creatinine (B), hemoglobin (C) and hematocrit (D) values throughout the experimental protocol. Results are presented as mean ± SEM: *- *p* < 0.05, **- *p* < 0.01, and ***- *p* < 0.001 *versus* Sham group. BUN, blood urea nitrogen.

The biochemical and hematological data for the Sham and CRF groups are presented in Table 2 and Fig 1. The CRF rats presented significantly (p<0.001) increased serum BUN and creatinine concentrations three weeks after the partial 5/6 nephrectomy. The values remained elevated until the 9^th^ week, after which a further increase was observed at the final time (p<0.001 and p<0.05, respectively), when compared with the Sham group (Fig 1A and 1B). Concerning the other biochemical parameters, we observed similar values, except for Total-c, ALT, hs-CPR and VEGF, which were significantly higher in CRF rats (p<0.05) at the final time (Table 2).

Three weeks after the 5/6 nephrectomy, the CRF rats developed anemia, as showed by the significant decline of Hb and HTC (p<0.001); the anemia persisted along the protocol. Analysing the results at the end of the protocol, we found that CRF animals showed significantly (p<0.01) decreased RBC count, and a trend towards a reduced reticulocyte count, when compared to the Sham rats. The MCV, MCH, RDW, PLT count and PDW values were similar and MCHC was significantly higher. Moreover, we found that WBC count, as well lymphocytes CD3^+^, CD3^+^CD4^+^, CD3^+^CD4^+^CD25^+^ and CD3^+^CD8^+^ percentages were similar for both groups. However, the percentage of activated cytotoxic T cells (CD3^+^CD8^+^CD25^+^) was significantly increased in the CRF group (p<0.01) *versus* Sham (Table 2).

### Serum EPO and liver and kidney EPO and EPOR mRNA expression

**Fig 2:**
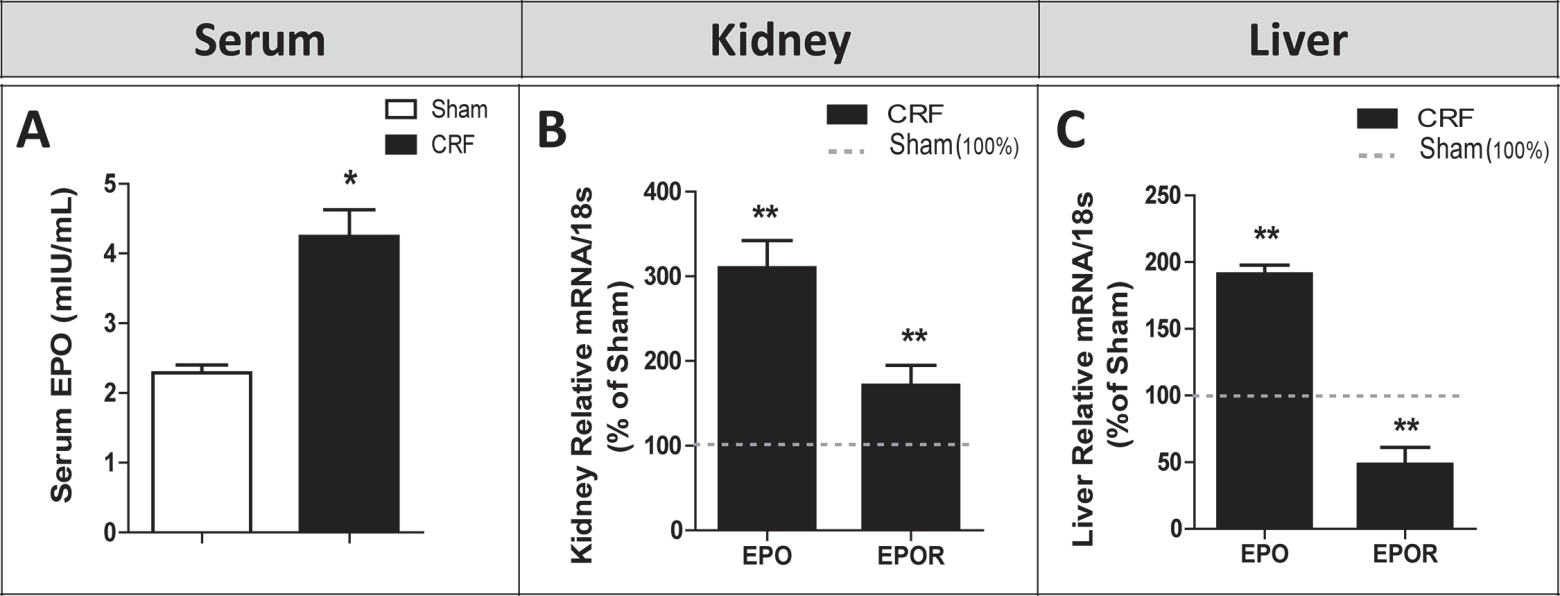
Serum EPO and kidney and liver gene expression of EPO and EPO receptor. Serum EPO (A), EPO and EPOR mRNA levels/18s expression (% of Sham group) in kidney (B) and liver (C) tissues, at the end of the study (12 weeks). Results are presented as mean ± SEM: *- *p* < 0.05, and **- *p* < 0.01 *versus* Sham group. EPO, erythropoietin.

At the final time, serum EPO was significantly higher (p<0.05) in the CRF group when compared with the Sham (Fig 2A). In addition, there was a significant (p<0.01) overexpression of EPO mRNA in the kidney and liver tissues of the CRF rats, when compared with the Sham animals (Fig 2B and 2C, respectively). Concerning EPOR mRNA, a significant (p<0.01) overexpression was found in the kidney tissue of the CRF rats, accompanied by a reduced (p<0.01) mRNA expression in the liver, when compared with the Sham group (Fig 2B and 2C, respectively).

### Iron metabolism

**Fig 3:**
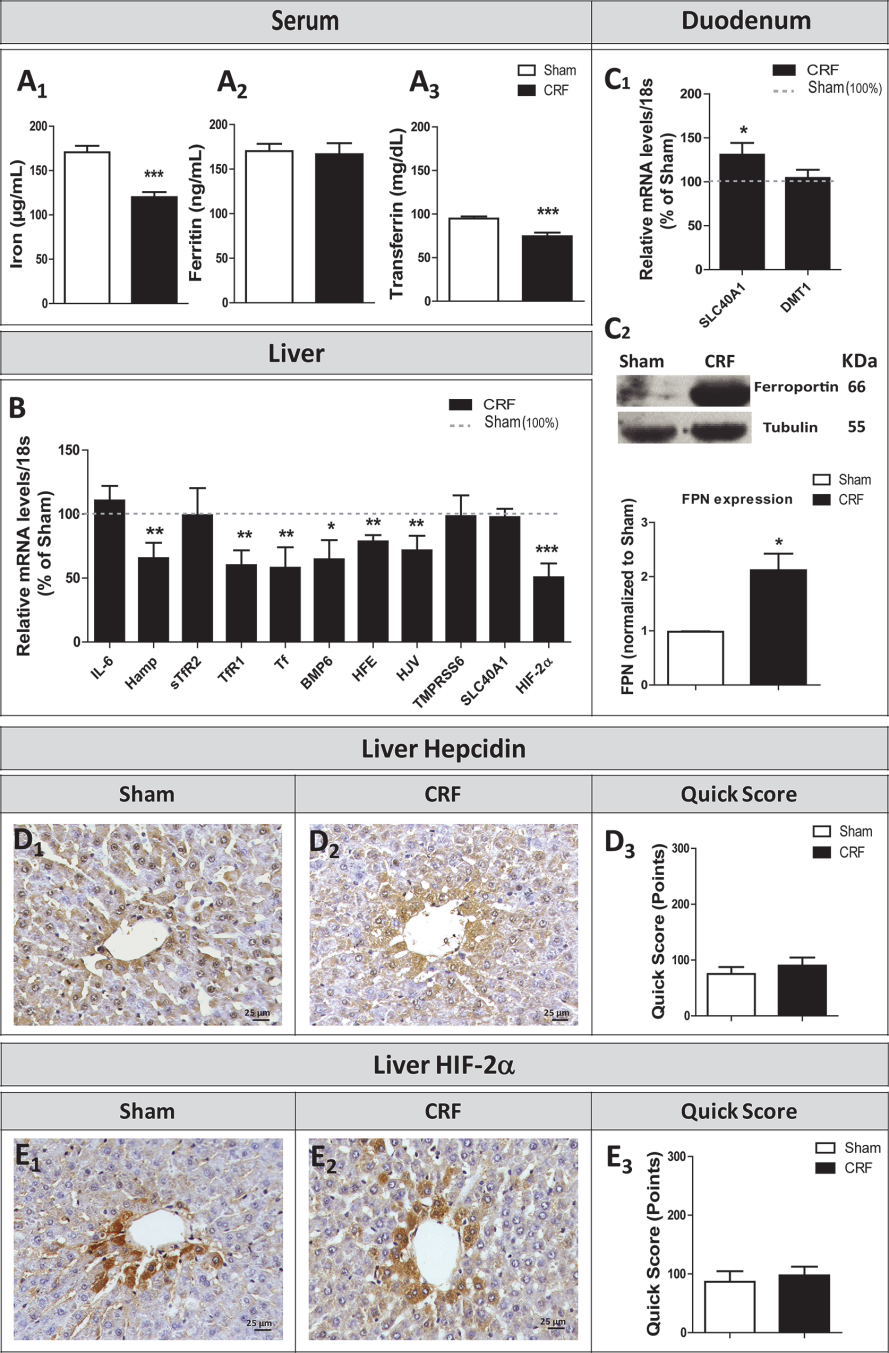
Serum iron (A_1_), ferritin (A_2_) and transferrin (A_3_) levels; relative gene expression mRNA levels/18s (% of Sham group) in liver (B) and duodenum (C) at final time (12 weeks). Immunohistochemical studies of the expression of Hepcidin (D_1_-D_3_) and HIF-2α (E_1_-E_3_) in the liver (original magnification, x400). Results are presented as mean ± SEM: *- *p* < 0.05, **- *p* < 0.01 and ***- *p* < 0.001 *versus* Sham group. BMP6, Bone morphogenic protein 6, DMT1, divalent metal transporter 1; Hamp, hepcidin antimicrobial peptide; HFE, Hemochromatosis; HIF-2α, Hypoxia inducible factor 2 alpha; HJV, Hemojuvelin; IL-6, interleukin-6; SLC40A1, ferroportin; TMPRSS6, Matriptase-2; sTfR2, soluble transferrin receptor 2; Tf, transferrin and TfR1, transferrin receptor 1.

To study iron metabolism we evaluated several markers at blood, tissue and cell (liver and duodenum) levels. When compared to sham group, we found a significant decrease in serum iron (p<0.001) and transferrin (p<0.001) in CRF rats (Fig 3A_1_ and 3A_3_), and similar values for serum ferritin (Fig 3A_2_).

No significant changes between groups were found for liver mRNA expression of *IL-6*, *sTfR2*, *TMPRSS6* and *SLC40A1* (Fig 3B); however, there was a significantly lower liver mRNA expression of *Hamp*, *sTfR1*, *TF*, *Hfe*, *HJV* (p<0.01 for all), *BMP6* (p<0.05) and *HIF-2α* (p<0.001) in the CRF rats when compared with the Sham ones (Fig 3B); in the duodenum, a significant mRNA overexpression (p<0.05) of *SLC40A1* and a similar mRNA expression of *DMT1* (Fig 3C_1_), were observed in CRF rats. In addition, the CRF rats presented a significant (p<0.05) duodenal overexpression of ferroportin (protein) when compared with the Sham animals (Fig 3C_2_). The immunohistochemical expression of liver hepcidin (Fig 3D_1_, 3D2 and 3D_3_) and of HIF-2α (Fig 3E_1_, 3E_2_ and 3E_3_) presented similar values for CRF and Sham rats.

**Fig 4:**
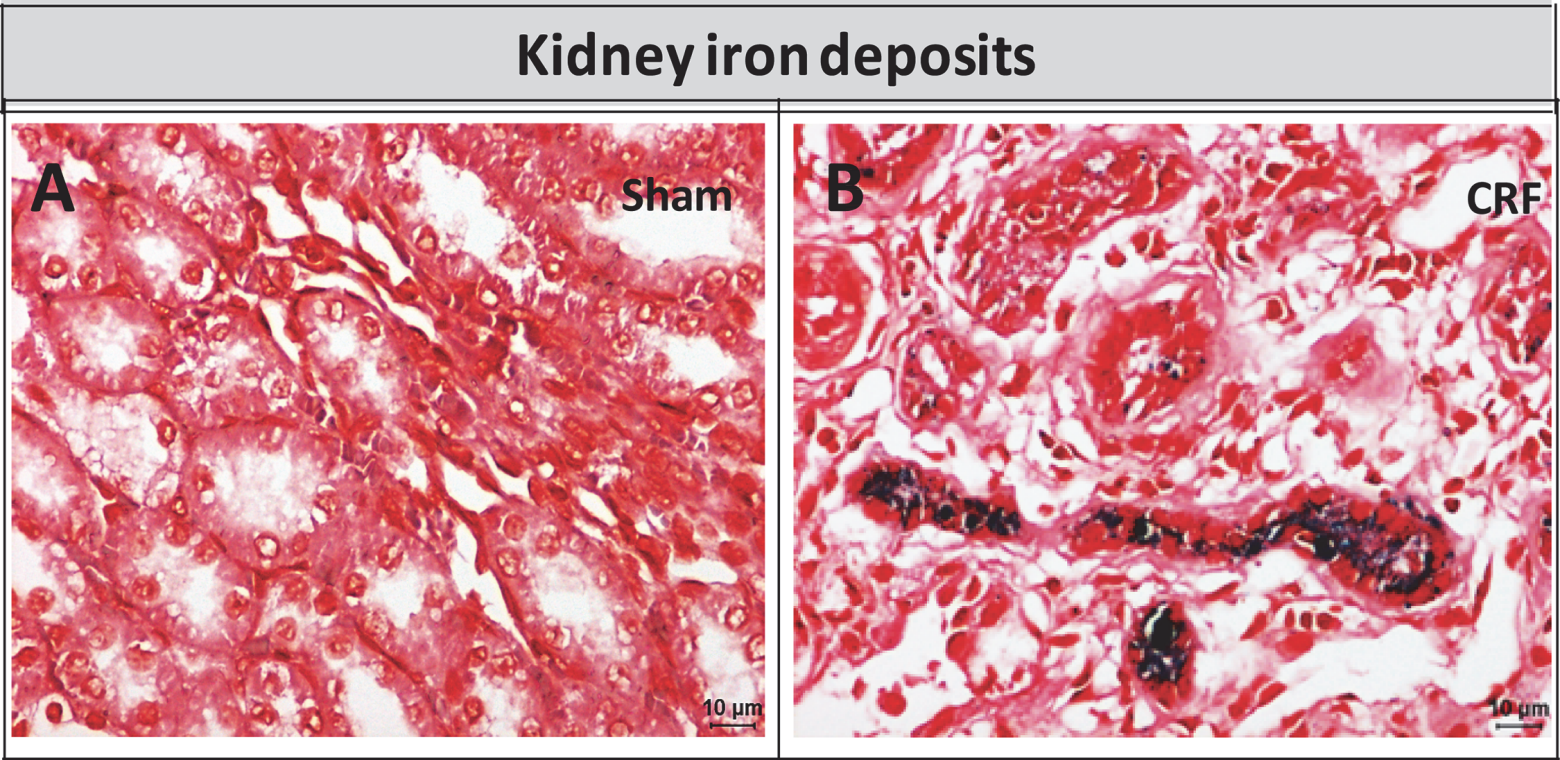
Perl’s staining in kidney of Sham (A) and CRF (B) rats. Original magnification, x400).

In the kidney tissue of CRF rats we found intense Perl’s staining, which is used as a marker of iron accumulation, contrarily to the absence of staining in kidneys of Sham rats (Fig 4).

### Kidney lesions

**Fig 5:**
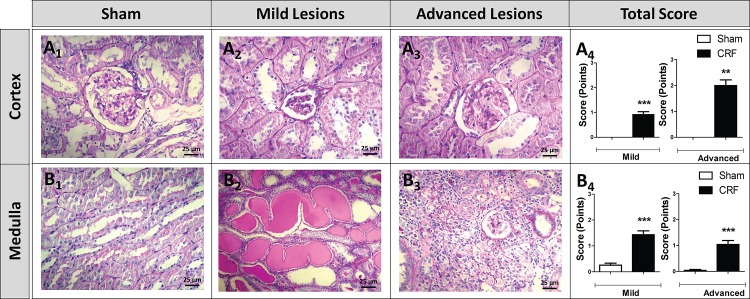
Glomerular and tubulointerstitial lesions. Representative glomerular (cortex) and tubulointerstitial (medulla) lesions observed in kidneys of CRF and Sham groups, at the final time (PAS staining): A_1_—normal glomerulus histology in the Sham rats; A_2_—glomerular atrophy and thickening of glomerular basement membrane; A_3_—glomerulus presenting mesangial expansion; A_4_—total score of mild and advanced glomerular lesions in both rat groups; B_1_—normal tubulointerstitial histology in the Sham rats; B_2_—hyaline cylinders; B_3_—interstitial fibrosis and tubular atrophy (IFTA); B_4_—Total score of mild and advanced tubulointerstitial lesions in both rat groups. Results are presented as mean ± SEM: **- *p* < 0.01 and ***- *p* < 0.001 *versus* Sham group.

No significant histomorphological changes were found in the kidneys of Sham rats at the end of the experimental period (Fig 5A_1_ and 5B_1_, Tables 3 and 4). However, the CRF rats presented several glomerular (cortex) and tubulointerstitial (medulla) lesions. Concerning the mild glomerular lesions, most of the animals of the CRF group presented thickening of Bowman´s capsule (score: 1.57±0.43; p<0.01), hyalinosis of vascular pole (score: 0.86±0.14; p<0.01), glomerular atrophy (score: 0.86±0.14; p<0.01) and hypercellularity (score: 1.00±0.00; p<0.01), while in Sham rats the lesions were absent (Table 3 and Fig 5). In addition, all CRF rats presented at least one of the advanced glomerular lesions, namely, mesangial expansion that was present in 5 out of 7 rats (Table 3 and Fig 5A_3_). The total score of both mild (0.91±0.12; p<0.001) and advanced (2.00±0.22; p<0.01) glomerular lesions showed a significantly increased value in CRF rats (Table 3 and Fig 5A_4_).

**Table 3:** Scoring and distribution (%) of mild and advanced glomerular lesions in the groups under study.

**Mild Lesions**		**0 Absent**	**1 <25%**	**2 25–50%**	**3 >50%**	**Total Score**
**Thickening of Bowman´s Capsule**	Sham, n (%)	7 (100%)	0	0	0	0.00±0.00
	CRF, n (%)	1 (14.3%)	3 (42.9%)	1 (14.3%)	2 (28.6%)	1.57±0.43**
**Hyalinosis of the vascular pole**	Sham, n (%)	7 (100%)	0	0 (0%)	0 (0%)	0.00±0.00
	CRF, n (%)	1 (14.3%)	6 (85.7%)	0	0	0.86±0.14**
**Glomerular atrophy**	Sham, n (%)	7 (100%)	0	0	0	0.00±0.00
	CRF, n (%)	1 (14,3%)	6 (85.7%)	0	0	0.86±0.14**
**Hypercellularity**	Sham, n (%)	7 (100%)	0	0	0	0.00±0.00
	CRF, n (%)	0	7 (100%)	0	0	1.00±0.00**
**Dilatation of the Bowman’s Space**	Sham, n (%)	7 (100%)	0	0	0	0.00±0.00
	CRF, n (%)	5 (71.4%)	2 (28.6%)	0	0	0.29±0.18
**Total Group Score**	Sham					**0.00±0.00**
	CRF					0.91±0.12***
**Advanced lesions**	**None of the previous (0)**	**Thickening of GBM(1)**	**Mesangial expansion(2)**	**Nodular sclerosis(3)**	**Global Glomerulosclerosis (4)**	**Total Score**
Sham, n (%)	7 (100%)	0	0	0	0	**0.00±0.00**
CRF, n (%)	0	1 (14.3%)	5 (71.4%)	1 (14.3%)	0	2.0±0.22**

Results are presented as mean ± SEM: *- *p* < 0.05

**- *p* < 0.01, and

***- *p* < 0.001 *versus* Sham group.

**Table 4:** Scoring and distribuition (%) of mild and advanced tubulointerstitial lesions in the groups under study.

**Mild Lesions**		**0 Absent**	**1 <25%**	**2 25–50%**	**3 >50%**	**Total Score**
**Tubular Hyaline Droplets**	Sham, n (%)	7 (100%)	0	0	0	0.00±0.00
	CRF, n (%)	0	7 (100%)	0	0	1.00±0.00 **
**TBM Irregularity**	Sham, n (%)	7 (100%)	0	0	0	0.00±0.00
	CRF, n (%)	0	3 (42.9%)	2 (28.5%)	2 (28.5%)	1.86±0.34**
**Tubular Dilatation**	Sham, n (%)	7 (100%)	0	0	0	0.00±0.00
	CRF, n (%)	2 (28.5%)	1 (14.3%)	2 (28.5%)	2 (28.5%)	1.57±0.48*
**Interstitial Inflammatory Infiltrate**	Sham, n (%)	4 (57.2%)	3 (42.9%)	0	0	0.29±0.18
	CRF, n (%)	0	0	7 (100%)	0	2.00±0.00**
**Vacuolar Tubular Degeneration**	Sham, n (%)	0	7 (100%)	0	0	1.00±0.00
	CRF, n (%)	3 (42.9%)	3 (42.9%)	1 (14.3%)	0	0.71±0.29
**Total Group Score**	Sham					**0.26±0.08**
	CRF					1.43±0.15***
**Advanced Lesions**		**0-Absent**	**1- <25%**	**2–25–50%**	**3- >50%**	**Score**
**Hyaline cylinders**	Sham, n (%)	6 (85.7%)	1 (14.3%)	0	0	0.14±0.14
	CRF, n (%)	0	1 (14.3%)	6 (85.7%)	0	1.86±0.14**
**Tubular Calcification**	Sham, n (%)	7 (100%)	0	0	0	0.00±0.00
	CRF, n (%)	7 (100%)	0	0	0	0.00±0.00
**Necrosis**	Sham, n (%)	7 (100%)	0	0	0	0.00±0.00
	CRF, n (%)	2 (28.6%)	5 (71.4%)	0	0	0.71±0.18*
**IFTA**	Sham, n (%)	7 (100%)	0	0	0	0.00±0.00
	CRF, n (%)	0	3 (42.9%)	4 (57.1%)	0	1.57±0.20**
**Total Group Score**	Sham					**0.04±0.04**
	CRF					1.04±0.16***

Results are means ± SEM

*- *p* < 0.05

**- *p* < 0.01, and

***- *p* < 0.001 versus the Sham group.

Concerning the mild tubulointerstitial lesions, most of the animals of the CRF group presented tubular hyaline droplets (score: 1.00±0.00; p<0.01), TBM irregularity (score: 1.86±0.34; p<0.01), tubular dilatation (score: 1.57±0.48; p<0.05) and interstitial inflammatory infiltration (score: 2.00±0.00; p<0.01) (Table 4 and Fig 5). Considering advanced tubulointerstitial lesions, the formation of hyaline cylinders (score: 1.86±0.14; p<0.01) and IFTA (score: 1.57±0.20; p<0.01) were the most relevant lesions observed in CRF rats, when compared with Sham rats (Table 4 and Fig 5B_2_ and 5B_3_). Once again, total score of both mild (1.43±0.15; p<0.001) and advanced (1.04±0.16; p<0.001) tubulointerstitial lesions showed a significantly increased value in the CRF rats (Fig 5B_4_).

### Renal expression of hypoxia inducible factors and other markers of kidney lesion

**Fig 6:**
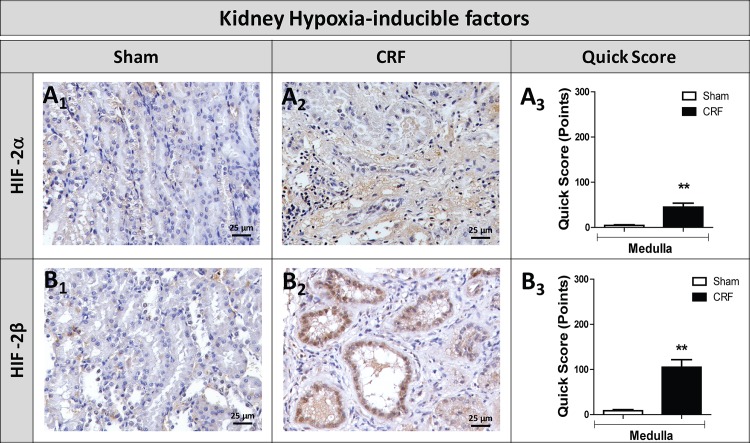
HIF-2α and HIF-2β immunohistochemical expression in the kidney. Kidney immunohistochemical expression of HIF-2α (A) and HIF-2β (B) in the renal cortex of sham (1) and CRF (2) rats and corresponding Quick scores (3). Results are presented as mean ± SEM: ***- *p* < 0.001 *versus* Sham group. HIF-2α, hypoxia inducible factor 2 alpha, and HIF-2β, hypoxia inducible factor 2 beta.

**Fig 7:**
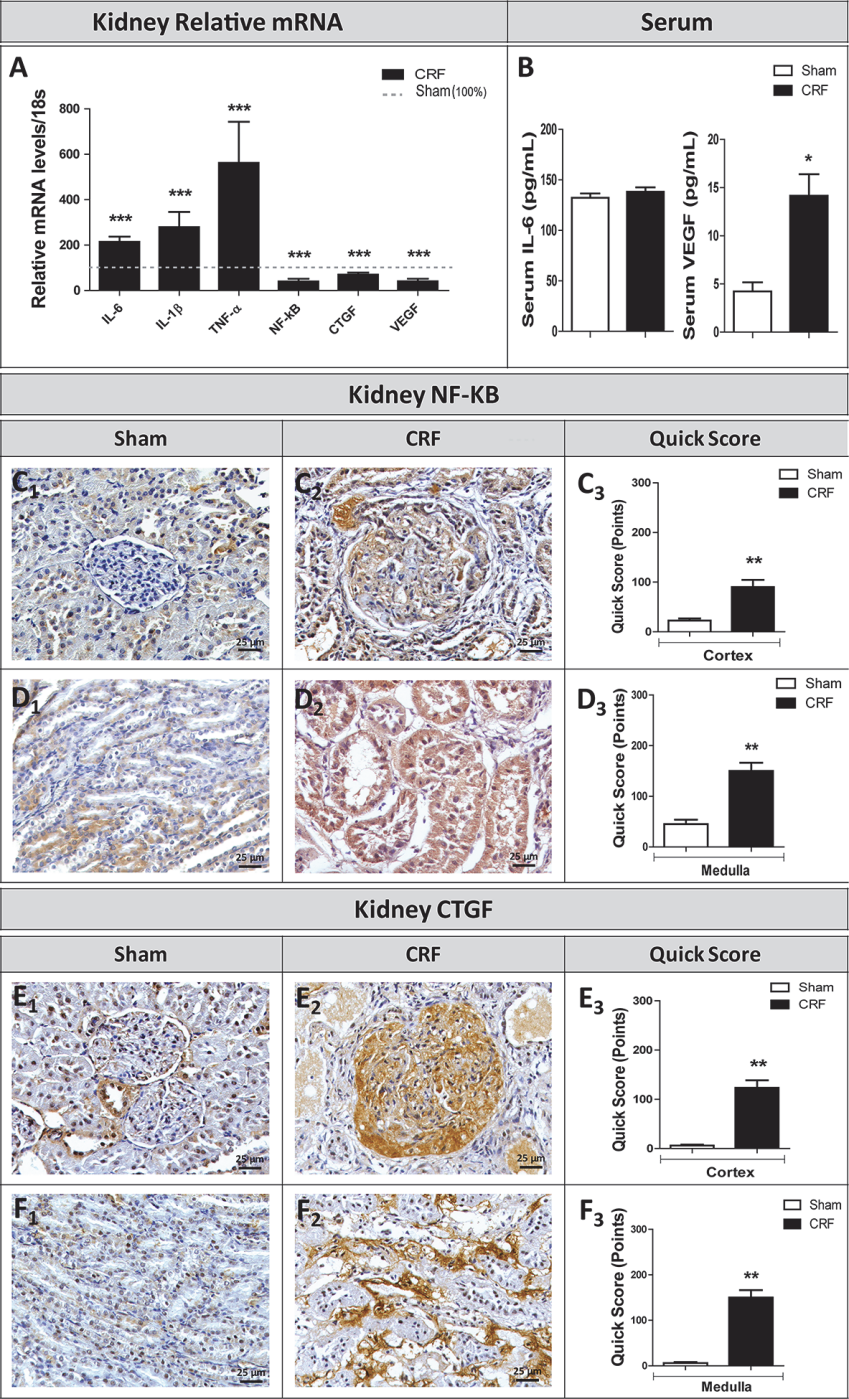
Relative gene expression mRNA levels/18s (% of Sham group) of markers of kidney lesions (A). Serum IL-6 and VEGF-α levels (B). Immunohistochemical expression of NF-kB and CTGF in renal cortex (C_1_-C_3_ and E_1_-E_3_, respectively) and medulla (D_1_-D_3_ and F_1_-F_3_, respectively) (original magnification, x400). Results are presented as mean ± SEM: *- *p* < 0.05 and **- *p* < 0.01 *versus* Sham group. CTGF, connective tissue growth factor; IL-1β, interleukin- 1 beta; IL-6, interleukin-6; NF-kB; nuclear transcription factor kappa B; TNF-α, tumor necrosis factor alpha; VEGF, vascular endothelial growth factor.

The histochemicals studies showed a significant increase (p<0.01) in the expression of *HIF-2α* and *HIF-2β* in the kidney of CRF rats, as compared to Sham rats (Fig 6A and 6B, respectively). In addition, we found a significant overexpression of *IL-6*, *IL-1β* and *TNF-* mRNA in the remnant kidney of CRF rats, when compared with Sham rats (p<0.001); contrarily, a significant downexpression of *NF-kB*, *CTGF* and *VEGF* (p<0.01) was found in the remnant kidney of CRF rats, when compared with Sham rats (p<0.001) (Fig 7A). Serum values for IL-6 were similar for both groups, while a significantly higher concentration for *VEGF* was observed in the CRF rats (p<0.05) (Fig 7B).

Concerning protein expression of NF-kB in the kidney tissue, we found a significantly higher immunoreactivity in the cortex and in the tubular epithelial cells (medulla) of the CRF rats, when compared with Sham ones (p<0.01) (Fig 7C_2_ and 7D_2_, respectively).

CTGF was weakly expressed in the glomerular (cortex) and interstitial cells (medulla) of Sham rat’s kidneys (Fig 7E_1_ and 7F_1_, respectively), while a significantly increased (p<0.01) expression of CTGF was noted in the glomeruli and interstitial cells (medulla) of the CRF rats (Fig 7E_2_ and 7F_2_, respectively).

## Discussion

Chronic kidney disease (CKD) is a general term for heterogeneous disorders affecting the structure and function of the kidney. The variation in the clinical pattern of the disease has been associated, to its etiology, severity and rate of progression. Since the introduction of the conceptual model, definition, staging of CKD and establishment of the clinical practice guidelines to treat kidney disease patients, the disease evolved from a life-threatening disorder affecting few people who needed care by nephrologists, to a common disorder of varying severity that deserves the attention of a multidisciplinary team and needs a concerted public health approach for prevention, early detection and management [27,28].

Early detection of renal failure and initiation of treatment contribute to prevent or delay some of these associated adverse effects [29]. Anemia, one of the most common complications of CKD, develops in the early phases of the disease, increasing its severity as the disease progresses, contributing to a poor quality of life of the patients [30]. Anemia is mainly associated with a reduced production of EPO by the failing kidneys and with disturbances in iron metabolism. However, the clear relationship between renal and extra-renal EPO production, iron deficiency, hypoxia and evolution of kidney lesions remain to be elucidated.

Animal models of CKD have been used as a tool to study the pathophysiological mechanisms underlying different stages of renal disease and of the associated anemia, as well as to test the efficacy of different therapies. The (5/6) nephrectomy model is the most used model of CKD, although there are different ways to achieve (5/6) reduction of nephron mass [29,31]. This model involves substantial removal of nephrons, followed by compensatory renal hypertrophy of the remnant kidney. Increasing workload by the remaining nephrons leads to progression of renal injury, namely to CKD [32–35].

The results of the present study confirmed that the surgical (5/6) nephrectomy model of CKD produced a sustained stage of renal insufficiency, as shown by the significantly increased BUN and creatinine concentrations, after three weeks of the surgical procedure (Fig 1); these values persisted for more 6 weeks, after which a further increment was observed, at the 12^th^ week, the end of protocol. In addition, a significant increase in kidney weight (KW) and in KW/BW ratio were found in CRF rats (Table 2), showing a compensatory renal proliferation/hypertrophy of the remnant kidney, as previously described for this model [36]. A trend towards an increase in heart weight (HW) and a significant increase of HW/BW ratio was also found, suggesting the development of left ventricle hypertrophy, which is a cardiac complication found in the CKD patients. In fact, besides the anemia secondary to renal insufficiency, CKD patients usually develop cardiac failure that further aggravates renal disease. This triad of dysfunctions, known as cardio-renal anemia syndrome, is responsible for the serious complications observed in these patients [37,38]. The progression of kidney disease and its associated cardiac/cardiovascular complications are the major causes of morbidity and mortality in these patients. One of the most prevalent co-morbidities is hypertension, which is present at all stages of CKD [39]. Our results confirmed the development of systolic hypertension, which is a typical feature in CKD rat model (Table 2). Usually, hypertension is inversely proportional to the residual functional renal mass, as occurs in human pathology [40,41].

As widely occurs in human CKD, we also observed the development of anemia, secondary to renal mass reduction, as shown by the reduced Hb and HTC values in the CRF rats, three weeks after nephrectomy that persisted along the following 9 weeks (Table 2). Moreover, the reticulocyte count showed a trend towards reduced values, suggesting a reduced erythropoietic response to overcome the anemia. However, we found that serum EPO concentration was increased in CRF rats at the final time (T4), as compared to sham animals (Fig 2), suggesting that EPO production is not reduced in this model of CKD-associated anemia, and that the remnant kidney or even extra-renal tissues were able to compensate EPO production. When peritubular fibroblasts in the kidney sense reduced oxygen tension, the production of hypoxia inducible factor (HIF) is induced, via oxygen sensitive prolyl hydroxylases (PHDs), triggering the activation of hypoxia response genes, leading to an increase in the production of EPO [42]. In the present work, we found a notable overexpression of EPO mRNA and EPOR mRNA in the kidney tissue of the CRF rats, as compared to Sham rats, as well as, an increased expression of HIF-2 (α and β) in the kidney tissue (Fig 6), which have been suggested as the main regulators of EPO synthesis in the adulthood, as recently reviewed by Haase [42]. These results suggest that the reduction of Hb levels in CRF rats, by leading to a reduced oxygen kidney perfusion, induced renal EPO production in response to low oxygen tension. The kidney is not the only organ able to adapt to EPO production, in response to low oxygen tension [43,44]. Apparently, chronic hypoxia is also sensed by a HIF-dependent mechanism in which a nitric oxide (NO)-mediated redistribution of blood flow from inner organs to the skin is able to cause a secondary increase in renal EPO production [45]. Extra-renal EPO production has been also described in the liver, although its contribution to the circulating EPO concentration in adults is highly debatable, even in kidney disease states [44]. Actually, we found a significantly increased liver EPO mRNA expression in CRF rats (Fig 2), accompanied by a reduced expression of EPOR mRNA. In spite of the increased serum EPO levels found in CRF rats, anemia persisted in these animals throughout the protocol, as well as the reticulocyte production, suggesting a blockade and/or a reduction in the activity of EPO. This hypothesis is supported by the fact that, when we administered rHuEPO (200 IU/Kg/week) to these CRF rats, a rise in hemoglobin level was observed, reaching similar or even higher values than those found in the sham group (data not shown). Actually, this response to the erythropoietic stimuli by exogenous recombinant human erythropoietin (rhEPO) further strengthens the hypothesis that the endogenous EPO, in spite of its higher plasmatic concentration, is not able to overcome anemia. The persistence of the anemia, might be explained by iron disturbances (Fig 3) and/or by a reduced (altered) EPO activity, and both changes may result from the glomerular kidney lesions (Fig 5) and from the developed local inflammatory milieu, as showed by the increased gene expression of different protein mediators of inflammation, hypoxia and fibrosis in the remnant kidney (Figs 6 and 7). Further studies should clarify the impact of kidney lesion on EPO structure and biological activity, namely the possibility of changes on glycosylation; however, current knowledge from the literature could support such hypothesis. In fact, following the translation of the *EPO* gene, three *N*-linked and one *O*-linked carbohydrate chains are added to erythropoietin; these chains normally exhibit heterogeneity in the type of carbohydrate moieties incorporated, chain length and branching configuration [46,47]; healthy individuals may present up to four residues on each *N*-linked carbohydrate chain, or up to two residues on the *O*-linked chain. Indeed, a variability in sialic acid composition may [48] affect the circulating half-life of erythropoietin and the interactions with its receptor; in general, increasing sialic acid content correlates with longer and greater potency of EPO [49]. Although the current understanding is, probably, incomplete, it is known that erythropoietin gene (*EPO*) expression is tightly regulated by several stimulators, namely, hypoxia-inducible transcription factors (HIF) and hepatocyte nuclear factor 4α, and by several inhibitors, including nuclear factor kappa B and GATA2 [50].

Iron is essential for the production of mature red blood cells and a normal iron metabolism is crucial to maintain body iron levels [10,51,52]. A disturbance in iron homeostasis is a hallmark of the anemia of CKD patients, which, usually, presents as a functional iron deficient anemia, with low serum iron and transferrin alongside with normal or even high ferritin [53]. In accordance, we found that CRF rats, as compared to Sham rats, showed a significant decrease in serum iron and transferrin levels and similar values for ferritin (Fig 3). In CKD patients the functional iron deficient anemia is explained by the underlying inflammatory process, with increased hepcidin levels. [13]. In this animal model, a systemic inflammatory state cannot be recognized, as showed by CRP and IL-6 serum values that are similar for the two groups (Table 2). However, a local renal inflammation, as suggested by the increased expression of IL-6, IL-1β and TNF-α mRNA in the kidney tissue (Fig 7), might contribute to alter EPO renal production/function and, therefore, erythropoiesis.

The evidence for hypoxic regulation of *Hamp* remains controversial. Some studies show that *Hamp* is suppressed by hypoxia through HIF-1- and (possibly) HIF-2-dependent pathways [15,54–56]. Indeed, *Hamp* contains some HREs in its promoter region and its expression might be reduced directly by hypoxia [15]. The evidence for this, however, is conflicting, as a recent study showed that HIF-1α and HIF-2α knockdown failed to reverse human *Hamp* repression by hypoxia; in addition, inducers of HIF (CO, hypoxia, oxalylglycine) also showed controversial results [57]. Furthermore, deletion of putative HREs in the human *Hamp* promoter did not alter its response to hypoxia. Choi et al. [58] also showed that the hypoxic downregulation of *Hamp* was independent of HIF-1α overexpression or knockdown; however, they suggested that the suppression of *Hamp* during hypoxia may involve HIF-2α. Our results are in accordance with this, as we observed a markedly increased kidney expression of HIF-2 in renal medulla of CRF rats (Fig 6). It is known that the expression of *Hamp* is modulated through several hepatocyte cell-surface proteins including Hfe, TfR2, HJV, TMPRSS6 and IL-6R. Regardless of the underlying molecular mechanism of reduction of *Hamp* expression during hypoxia, a decrease in hepcidin leads to an increased iron uptake and absorption at the duodenum, as well as to an increased iron release from the macrophages, favoring Hb synthesis and erythropoiesis [16]. Actually, we found a significant downregulation in liver mRNA expression of *Hamp*, *sTfR1*, *TF*, *Hfe*, *HJV* and *BMP6* in CRF rats, as compared with the Sham rats (Fig 3), together with a significant duodenal overexpression of ferroportin gene (codified by *SLC40A1*) and protein in CRF rats and similar values for the expression of *DMT1* gene (Fig 3), suggesting that iron absorption in the enterocytes was normal or even enhanced, as the increase in duodenal ferroportin expression may occur to counteract the low iron levels observed in CRF rats; in addition, hypoxia might contribute to the significantly decreased liver *Hamp* mRNA expression in CRF rats. Likewise, the absence of inflammation in CRF rats, as showed by normal values of serum and liver IL-6 expression, as well as of reduced serum hs-CRP, are in agreement with lower liver *Hamp* mRNA expression in these animals.

This model of CKD has been associated to glomerulosclerosis and progressive tubulointerstitial damage. Although the mechanisms of tubular injury are poorly clarified in this and in other models of renal disease, proteinuria has a crucial role [59]. It has been proposed that filtered iron may have also a role in tubular injury, when associated with proteinuria. Actually, in proteinuric states, as a result of the glomerular leak of transferrin, iron might be released from transferrin in the acid milieu of the tubular lumen [59]. In fact, iron accumulation is observed in the proximal tubule in human CKD [60], as well as in rat models with nephropathy [59,61,62] and seems to be associated with the progression of CKD. By performing Perl’s staining of kidney slides, to search for iron accumulation within rat kidney tubules, we found that iron deposits were almost undetectable in Sham rats and were increased in CRF rats (Fig 4), suggesting that the leakage of iron through damaged glomerulus may explain the reduced serum iron and transferrin observed in CRF rats. Actually, considering the anemia in the absence of systemic inflammation, a rise in serum iron would be expected to face the needs for erythropoiesis. Naito et al. [63] studied the effect of dietary iron restriction on the renal damage developed in a rat model of CKD, presenting nephron hyperfiltration, glomerulosclerosis and tubulointerstitial injury, and found that iron restriction attenuated these changes in CKD rats. This beneficial effect of iron restriction on renal damage is consistent with the results previously reported in the different models of renal disease [64,65].

It is widely accepted that, regardless of the initial cause of renal failure, tubulointerstitial fibrosis is the major cause of disease progression in CKD [66,67]. Typically, the functional impairment in CKD correlates with tubulointerstitial fibrosis, and with glomerulosclerosis. Tubulointestitial damage is closely correlated with reduced creatinine clearance and is currently the best predictor of disease progression [67]. Hypoxia and altered O_2_ perfusion are also potential players in the development of renal injury [67]. As referred, in response to low oxygen supply, hypoxia-inducible factors (HIFs) are produced triggering the expression of the hypoxia response genes, increasing the production of EPO, VEGF and of glycolytic enzymes. It is unclear if the increase in HIF has a renoprotective role, or if it contributes to interstitial fibrosis and/or tubular atrophy. This duality of effects has also been described for vascular endothelial growth factor (VEGF), another target gene of HIFs [18]. We found significantly high serum VEGF levels (almost three fold the control value) (Fig 7), probably explaining the repression in VEGF gene expression in the remnant kidney of CRF. Under hypoxic conditions in renal injury, the HIF system is activated, even before any histological evidence of tubulointerstitial damage [68,69], and the degree of HIF expression seems to correlate with the extent of tubular injury. However, whether this increased activity is beneficial or harmful is unclear and may well depend on the context, the cell type affected and/or the duration of HIF expression. Another major target gene for HIF is the pro-fibrotic connective tissue growth factor (CTGF) [70]. In our study, CRF rats presented several glomerular and tubulointerstitial lesions (Fig 5). Mild glomerular lesions were observed in most of the CRF rats, presenting thickening of Bowman capsule, hyalinosis of vascular pole, glomerular atrophy and hypercellularity (Table 3). All CRF rats presented at least one advanced glomerular lesion; mesangial expansion was the more frequent lesion. Regarding the mild tubulointerstitial lesions, most of the CRF rats presented tubular hyaline droplets, TBM irregularity, tubular dilatation and interstitial inflammatory infiltration. Concerning advanced tubulointerstitial lesions, hyaline cylinders and IFTA were the most relevant lesions observed in CRF rats. As referred, besides hypoxia, CRF rats showed local inflammation in the remnant kidney, as suggested by overexpression of *IL-6*, *IL-1β* and *TNF-* genes, as well as NF-kB, a key mediator of inflammation. In addition, we found an overexpression of CTGF in the glomeruli and in interstitial cells (medulla), in agreement with the existence of tubulointertitial lesions and fibrosis.

**Fig 8:**
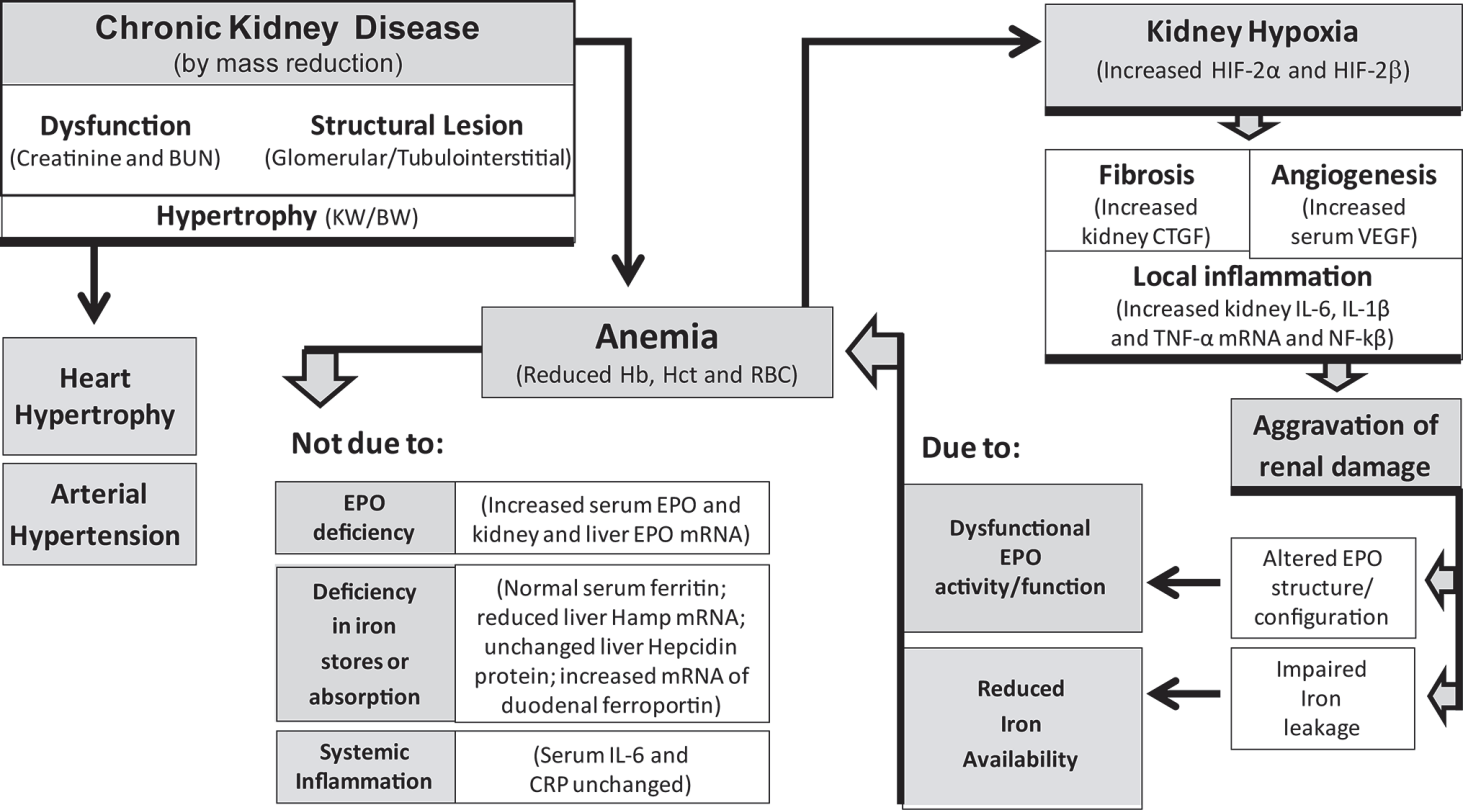
Schematic diagram representing a putative mechanistic model of the relationship between CKD, anemia, EPO production, iron/hepcidin metabolism, inflammation, hypoxia and renal damage/fibrosis in the remnant kidney rat model.

In summary (Fig 8), we found that this model of CKD induced by 5/6 nephrectomy presented a sustained degree of renal dysfunction with mild and advanced glomerular and tubulointerstitial lesions. Anemia developed early after nephrectomy and persisted throughout the study. However, the remnant kidney was still able to produce EPO and the liver seems to increase EPO production. In spite of the increased EPO blood levels, circulating reticulocytes did not increase and, therefore, the anemia did not improve. The persistence of anemia may result from a dysfunctional EPO or from reduced iron availability, as suggested by the low serum iron and transferrin levels. Despite the increased expression of duodenal ferroportin in the CRF rats, favouring iron absorption, iron levels were reduced, which might be due to iron leakage caused by advanced glomerular and tubular kidney damage. Our data also suggest that the anemia of CKD and the associated kidney hypoxia favour the development of fibrosis, angiogenesis and a local inflammatory milieu in the kidney that seem to underlie a “resistance” to EPO stimuli and reduced iron availability. These findings might contribute to open new windows to identify putative therapeutic targets for this condition, as well as for rHuEPO resistance, which occurs in 5–10% of CKD patients.
